# Firearm purchasing and firearm violence during the coronavirus pandemic in the United States: a cross-sectional study

**DOI:** 10.1186/s40621-021-00339-5

**Published:** 2021-07-05

**Authors:** Julia P. Schleimer, Christopher D. McCort, Aaron B. Shev, Veronica A. Pear, Elizabeth Tomsich, Alaina De Biasi, Shani Buggs, Hannah S. Laqueur, Garen J. Wintemute

**Affiliations:** 1 Violence Prevention Research Program, Department of Emergency Medicine, University of California, Davis, Sacramento, CA USA; 2 University of California Firearm Violence Research Center at UC Davis, 2315 Stockton Blvd, Sacramento, CA 95817 USA

**Keywords:** Firearm, Gun, Violence, Domestic violence, Coronavirus, COVID-19

## Abstract

**Background:**

Firearm violence is a significant public health problem in the United States. A surge in firearm purchasing following the onset of the coronavirus pandemic may have contributed to an increase in firearm violence. We sought to estimate the state-level association between firearm purchasing and interpersonal firearm violence during the pandemic.

**Methods:**

Cross-sectional study of the 48 contiguous states and the District of Columbia from January 2018 through July 2020. Data were obtained from the National Instant Criminal Background Check System (a proxy for firearm purchasing) and the Gun Violence Archive. Using negative binomial regression models, we estimated the association between cumulative excess firearm purchases in March through July 2020 (measured as the difference between observed rates and those expected from autoregressive integrated moving average models) and injuries (including nonfatal and fatal) from intentional, interpersonal firearm violence (non-domestic and domestic violence).

**Results:**

We estimated that there were 4.3 million excess firearm purchases nationally from March through July 2020 and a total of 4075 more firearm injuries than expected from April through July. We found no relationship between state-level excess purchasing and non-domestic firearm violence, e.g., each excess purchase per 100 population was associated with a rate ratio (RR) of firearm injury from non-domestic violence of 0.76 (95% CI: 0.50–1.02) in April; 0.99 (95% CI: 0.72–1.25) in May; 1.10 (95% CI: 0.93–1.32) in June; and 0.98 (95% CI: 0.85–1.12) in July. Excess firearm purchasing within states was associated with an increase in firearm injuries from domestic violence in April (RR: 2.60; 95% CI: 1.32–5.93) and May (RR: 1.79; 95% CI: 1.19–2.91), though estimates were sensitive to model specification.

**Conclusions:**

Nationwide, firearm purchasing and firearm violence increased substantially during the first months of the coronavirus pandemic. At the state level, the magnitude of the increase in purchasing was not associated with the magnitude of the increase in firearm violence. Increases in purchasing may have contributed to additional firearm injuries from domestic violence in April and May. Results suggest much of the rise in firearm violence during our study period was attributable to other factors, indicating a need for additional research.

**Supplementary Information:**

The online version contains supplementary material available at 10.1186/s40621-021-00339-5.

## Background

Firearm violence is among America’s leading causes of death and disability (Wintemute, 2015) and has profound adverse social, psychological, and economic effects (Ranney et al., 2019). A large body of research has established an association between firearm access and risk of interpersonal and self-directed firearm violence at the population (Miller et al., 2002), household (Kellermann et al., 1993; Kellermann et al., 1992), and individual (Wintemute et al., 1999; Studdert et al., 2020) levels. Surges in firearm purchasing, which occur after mass shootings and significant political events, have been associated with subsequent population-level increases in firearm violence (Laqueur et al., 2019; Levine & McKnight, 2017).

The coronavirus pandemic has created deep and widespread social and economic disruption in the United States (US). As of June 2021, over 33 million cases and approximately 600 thousand deaths have been reported (Johns Hopkins Coronavirus Resource Center, 2021). Federal Bureau of Investigation (FBI) records of background checks pursuant to firearm purchases (Federal Bureau of Investigation, 2021) suggest a substantial surge in firearm purchasing near the onset of the coronavirus pandemic. Given prior findings on the relationship between firearm violence and firearm access and surges in firearm purchasing specifically (Laqueur et al., 2019; Levine & McKnight, 2017), it is reasonable to expect a subsequent increase in firearm violence.

Other effects of the pandemic, or the country’s response to it, might modify the relationship between surges in firearm purchasing and firearm violence. Stay-at-home orders might reduce community violence since fewer people are in public places—or increase it if fewer potential witnesses are on scene or law enforcement presence is reduced. Violence at home might rise if stay-at-home orders intensify contact between persons in violent relationships (Bullinger et al., 2020). Any impact on violence due to stay-at-home orders might be limited to the time those orders are in effect (McKay et al., 2020).

The pandemic has also exacerbated factors that contribute to interpersonal violence—including financial stress, trauma, and strains on community resources—particularly among Black, Indigenous, and other communities of color (Sequist, 2020), which already experience a disproportionate burden of interpersonal firearm violence (Boeck et al., 2020). In addition, recent killings of Black people, and the broader racial inequities they reflect, have spurred nationwide protests and counter-protests that have been accompanied by violence. These events may heighten concerns about violence and contribute to an increase in firearm purchasing (Kravitz-Wirtz et al., 2021).

In this study, we estimated the association between changes in firearm purchasing and interpersonal firearm violence during the coronavirus pandemic and described whether and how the association evolved through July 2020. The present study extends our prior work, which found a positive association between firearm purchasing and firearm violence through May 2020 (Schleimer et al., 2020a). We hypothesized that the purchasing surge would be associated with an increase in firearm violence. Our overall aim was to provide evidence on the relationship between firearm purchasing and firearm violence during the coronavirus pandemic, with the ultimate goals of informing future research and firearm violence prevention strategies.

## Methods

### Design and setting

This was a cross-sectional study of monthly firearm purchasing and firearm violence in the US from January 2018 through July 2020. The 48 contiguous states and District of Columbia (DC) were included, resulting in 1519 place-time units (31 months × 48 states and DC). Hawaii and Alaska were excluded due to missing or incomplete data.

### Data and sources

We approximated firearm purchasing using state-level National Instant Criminal Background Check System (NICS) data (Federal Bureau of Investigation, 2020) specific to firearm purchase transactions (excluding checks for pawn redemptions or carry permits). Denominators for rates were obtained from the US Census’ Annual Estimates of the Resident Population (US Census Bureau, 2020). NICS checks do not have a 1:1 correspondence with purchased firearms because most states permit multiple firearm purchases in a single transaction; we assumed this discordance did not change differentially over both time and place, though data to validate this assumption are currently lacking.

Interpersonal firearm violence was measured with data from the Gun Violence Archive (GVA), which compiles records of gun violence from approximately 7500 news outlets and other public sources (Gun Violence Archive, n.d.). Data used here included the event’s date and location and selected characteristics. We included only events coded as intentional, interpersonal violence (i.e., assault) with 1 or more shots fired and 1 or more persons killed or injured. That is, we excluded events classified by GVA as unintentional (e.g., “accidental shooting,” or “thought gun was unloaded”) or self-directed (e.g., “suicide attempt,” “suicide by cop”). We use the term ‘injuries’ to include both nonfatal injuries and deaths.

We then separated interpersonal firearm violence events as domestic violence (DV) related and non-DV related. DV events were identified by GVA with an indicator (DV: yes or no). GVA’s classification of DV has not been validated (to our knowledge), and GVA does not provide a precise definition of DV (e.g., whether DV events can include violence among household or family members in addition intimate partners). GVA does, however, provide links to the original source of information (e.g., news article) for each event, and we reviewed a sample to gain a better understanding of the types of events GVA classified as DV. We found that some articles specified the relationship between the victim and suspect (usually current or former intimate partners or family members), while others were simply labeled as “domestic-related” or the result of a “domestic dispute,” for example. We classified all other (non-DV) events as non-domestic interpersonal firearm violence.

We developed a directed acyclic graph to identify potential confounders that should be adjusted for in analyses (Hernán et al., 2002) (Supplementary Fig. 1; data sources and variable details are in Supplementary Table 1; Additional file 1): COVID-19 cases and deaths; state stay-at-home orders and mobility measured with aggregated smartphone data (a measure of physical distancing, i.e., adherence to the orders); internet searches for a racial epithet, attendees at protests against racial injustice, and police violence during protests against the killing of George Floyd, all as proxies for racism and responses to it; baseline firearm purchasing rates; unemployment; temperature; and precipitation. We additionally considered changes to state background check laws to account for temporal variation in exposure measurement.

### Statistical analyses

Our primary exposure was excess firearm purchasing, measured as the difference between observed and expected monthly rates of firearm purchasing per 100,000 population following the onset of the pandemic. We estimated expected rates for each state-month between March and July 2020 with seasonal auto-regressive integrated moving average models (ARIMA) (Box & Jenkins, 1970) fit to training data beginning in January 2011 and ending in February 2020. Models were parameterized using the Hyndman and Khandakar algorithm (Hyndman et al., 2020), and residual autocorrelation was examined using the Box-Ljung test (Ljung & Box, 1978) with the Benjamini and Hochberg (Benjamini & Hochberg, 1995) correction for multiple testing. Forecast accuracy was assessed with mean squared error (MSE) computed from time-series cross-validation (Hyndman et al., 2020).

We measured excess purchases beginning in March and accumulated the excess for each month through July. We defined the exposure this way to account for variation between months while allowing for the accumulation of excess purchases over time, as the risk of violence associated with increased purchasing may not be immediate or time-limited.

We estimated the association between changes in firearm purchasing and firearm violence using multivariable unconditional negative binomial regression models. For our regressions, we rescaled the exposure to be 1 excess purchase per 100 population (rather than per 100,000 population) to improve interpretability of the strength of the association. Testing of the dispersion parameter (using likelihood ratio tests) confirmed that a negative binomial model was preferred to Poisson for both outcomes (*p* < 0.001). The outcomes were modeled as counts of firearm injuries (nonfatal and fatal) from non-domestic interpersonal violence, and separately, from domestic violence. We combined nonfatal and fatal injuries because both have significant impacts on health and the difference between a nonfatal injury and a fatal injury is arguably more a function of factors like the shooter’s aim and timely health care access (Crandall et al., 2013) than it is a function of firearm availability.

We used an event study analysis that allows for a dynamic association between excess purchasing and firearm violence over time. A series of coefficients describe the evolution of the association in each month from April 2020 through July 2020. Our model is as follows:

$$ \mathit{\log}\left({Y}_{iy m}\right)={\beta}_0+\sum \limits_{p=1}^4{\beta}_p{E}_{ip}+{\alpha}_{ym}+{\theta}_i+{\delta}_y+{\lambda}_m+\gamma {X}_{iy m}+\log \left({pop}_{iy}\right) $$

Where *Y*_*iym*_ is the count of firearm injuries in state i, year y and month m, and *β*_0_ is the intercept. Each *E*_*ip*_ is a vector of values which are equal to the estimate of cumulative excess purchasing when an observation is p months after March 2020 and 0 otherwise. *β*_*p*_ denotes a series of coefficients that describe the association between excess purchasing and firearm violence in each period, p, after the surge beginning in April 2020 and ending in July 2020. To mitigate potential bias arising from the possibility that changes in violence drove changes in purchasing, firearm violence in each month from April through July was predicted by the cumulative excess purchasing through the prior month. Models included a pre-post dummy for March 2020 (*α*_*ym*_ = 1 if March 2020 or later; 0 otherwise); indicators for states to control for time-invariant characteristics of states (*θ*_*i*_), and year (*δ*_*y*_) and month (*λ*_*m*_) to control for state-invariant secular and seasonal trends; and a set of time-varying covariates (*X*_*iym*_) that were identified a priori but retained according to model fit. Specifically, we forced four covariates in the models that we believed to be stronger confounders than others (coronavirus deaths and cases, mobility/physical distancing, unemployment, and baseline purchasing rates) and compared models with all possible combinations of the other covariates, selecting the model that minimized Akaike Information Criteria (AIC). Time-varying covariates were expressed as two-month moving averages (i.e., the average of the current month and the month prior). The log of the population, log(*pop*_*iy*_), was included as an offset, and coefficients were exponentiated and interpreted as rate ratios (RR).

The correlation between variables ranged from − 0.3 (for temperature and expected purchasing rates) to 0.8 (for physical distancing with unemployment and stay-at-home orders). The highest correlation with our exposure was 0.6 (with unemployment). We used Variance Inflation Factors (VIF) to assess the degree to which the variance in our parameter estimates was inflated by the correlation among the other independent variables in the model. We found little evidence of variance inflation, particularly for our exposure of interest (VIFs below 2). In light of this and epidemiologic guidance on the bias-variance tradeoff (Schisterman et al., 2017), we maintained variables in our models that we hypothesized, a priori, to be confounders.

To incorporate uncertainty from both stages of the estimation, we constructed confidence intervals (CIs) using a semi-parametric bias-corrected and accelerated bootstrap with 5000 repetitions. The bootstrap procedure sampled states with replacement, repeatedly simulated the time series to re-estimate expected and excess firearm purchasing rates taking into account model uncertainty and random error, and then re-computed the corresponding exposure coefficients.

### Additional and sensitivity analyses

Because physical distancing may affect where violence takes place and how many people are at risk, we also modeled outcomes as: 1) counts of events, rather than injuries; and 2) the ratio of injuries to events (using linear models).

To test the robustness of our findings, we first included state-specific linear trends to adjust for unmeasured confounders that are neither time- nor state-invariant. Second, to adjust for the influence of past violence on future purchasing, we included 2-month lagged values of the outcome as a predictor. While the combination of lagged dependent variable and state fixed effects can generate bias, this bias is inversely proportional to the number of time periods and is likely small in our case (Nickell, 1981). Third, we excluded the District of Columbia, because it is a city. Fourth, we additionally controlled for all-cause mortality (excluding deaths from interpersonal firearm violence) to capture misclassification of coronavirus deaths and broader consequences of the pandemic on population health. Finally, to test the sensitivity of estimates to covariates, we included all hypothesized confounders (Supplementary Fig. 1; Additional file 1) rather than the subset that minimized AIC.

We estimated the power of our main analysis using a Monte Carlo simulation (Arnold et al., 2011). To simplify the simulation, we assumed the effect size was the same for all four exposure coefficients. We estimated that we are sufficiently powered (1-β ≥ 80%) to detect a true RR of 1.3 for non-domestic firearm violence and a true RR of 1.7 for domestic firearm violence.

Analyses were done with the forecast package (version 8.12) in R, version 4.0.0 (R Foundation for Statistical Computing) and in Stata, version 15.1 (StataCorp).

## Results

### Descriptive

**Fig. 1:**
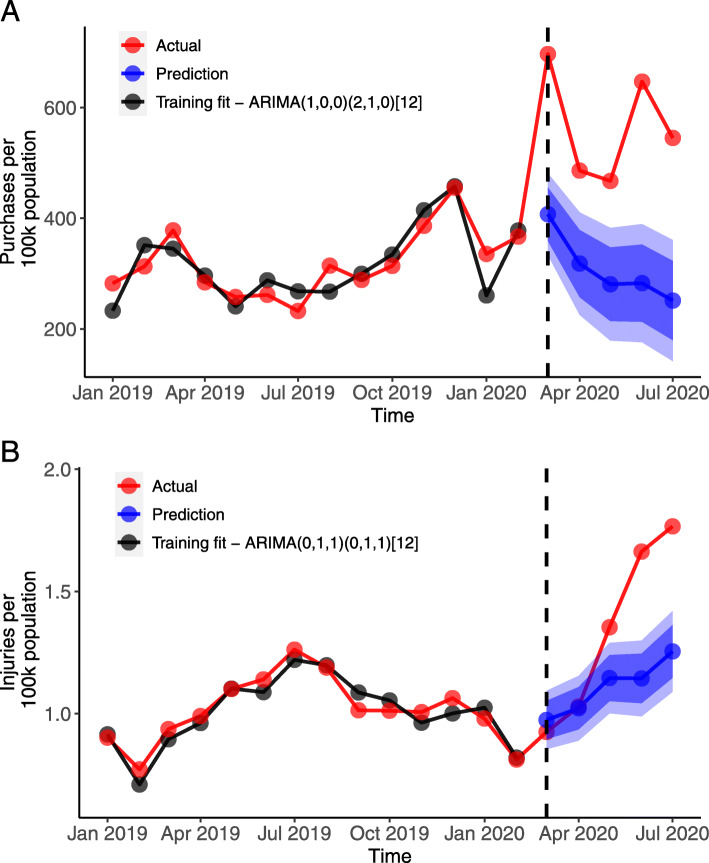
Nationwide Trends in Firearm Purchasing (Panel A) and Firearm Violence (Panel B). A) Monthly firearm purchases per 100,000 population, with training data from January 2011 through February 2020 (data source: FBI National Instant Criminal Background Check System). B) Monthly injuries (nonfatal and fatal) from intentional, interpersonal firearm violence per 100,000 population, with training data from January 2015 through February 2020 (data source: Gun Violence Archive). Dotted line indicates March 2020. Dark and light blue bands indicate 80 and 95% prediction intervals, respectively

**Fig. 2:**
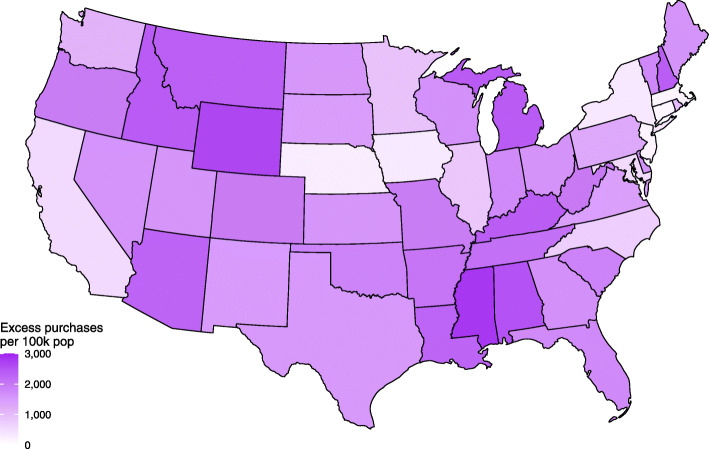
Map of Cumulative Excess Firearm Purchases By US State, March Through July 2020. Data source: FBI National Instant Criminal Background Check System

There was a sharp increase in firearm purchasing in the US in March 2020, and purchasing rates remained high through July (Fig. 1A). We estimated that there were 4.3 million excess firearm purchases nationally between March and July 2020, an excess rate of 1302 purchases per 100,000 population over the 5-month period and an 85% increase over expected volume. Across states from March through July, there was an average excess of 301.7 purchases per 100,000 population per month (Table 1), with substantial variability in cumulative excess purchasing rates by the end of the 5-month period ending in July 2020, ranging from 37.4 (DC) to 2804.8 (Mississippi) per 100,000 population (average: 1508.7) (Fig. 2). MSE was 1.7 on average (range: 1.2–3.4) for the forecast accuracy of state-level ARIMA estimates of expected firearm purchasing rates per 100,000.

**Table 1:** Description of Intentional, Interpersonal Firearm Violence and Excess Firearm Purchasing Pre- and Post-Purchasing Surge, 48 Contiguous US States and The District of Columbia, 2018–2020

	January 2018–February 2020	March 2020–July 2020
Monthly firearm injuries and deaths from non-domestic violence per 100,000 state population, mean (SD)	0.98 (0.95)	1.36 (1.60)
Monthly firearm injuries and deaths from domestic violence per 100,000 state population, mean (SD)	0.05 (0.06)	0.07 (0.08)
Monthly excess firearm purchases per 100,000 state population,^a^ mean (SD)	NA	301.7 (175.6)

^a^Based on model estimates from ARIMA models

*NA* not applicable, *SD* standard deviation

Nationally, interpersonal firearm violence increased substantially beginning in May 2020 (Fig. 1B). From April through July, the period used to measure the association between firearm purchasing and firearm violence, we estimated that there were 4075 more firearm injuries and deaths (from both non-domestic and domestic violence) than expected, reflecting a 27% increase. Prior to the purchasing surge (i.e., from January 2018 through February 2020), the average statewide monthly rate of firearm injuries from non-domestic violence was 0.98 per 100,000 population, and the average statewide rate for domestic violence-related firearm injuries was 0.05 per 100,000 population (Table 1). From March through July 2020, observed average monthly rates of non-domestic and domestic firearm injuries were 1.36 and 0.07 per 100,000, respectively.

### Firearm purchasing and non-domestic firearm violence

We found no association between state-level excess firearm purchasing and non-domestic firearm violence (Table 2). Results from the multivariable model indicated that, in April, each excess purchase per 100 population in the month prior was associated with a rate ratio of 0.76 (95% CI: 0.50, 1.02); in May, the RR for the prior 2 months was 0.99 (95% CI: 0.72, 1.25); in June, the RR for the prior 3 months was 1.10 (95% CI: 0.93, 1.32); and in July, the RR for the prior 4 months was 0.98 (95% CI: 0.85, 1.12).

**Table 2:** Association Between Cumulative Excess Firearm Purchasing and Interpersonal Firearm Violence, 2018–2020

	Unadjusted			Adjusted		
	RR	95% CI		RR	95% CI	
*Non-DV-related firearm violence*^*a*^						
1 excess firearm purchase per 100 population						
April	0.81	0.63	1.01	0.76	0.50	1.02
May	1.04	0.88	1.22	0.99	0.72	1.25
June	1.17	1.00	1.35	1.10	0.93	1.32
July	1.14	1.03	1.25	0.98	0.85	1.12
*DV-related firearm violence*^*b*^						
1 excess firearm purchase per 100 population						
April	1.66	0.94	3.00	2.60	1.32	5.93
May	1.47	1.14	1.92	1.79	1.19	2.91
June	1.21	0.90	1.70	1.03	0.66	1.52
July	1.09	0.94	1.29	0.89	0.66	1.12

Results are from negative binomial regression models with the log of the population as an offset. All models include indicators for states, year, and month

^a^The outcome is counts of non-domestic violence-related firearm injuries (nonfatal and fatal). Adjusted estimates are from a model that additionally includes: a pre-post dummy for March 2020, COVID-19 cases and deaths, mobility, unemployment, baseline firearm purchasing rates, police violence during the George Floyd protests, stay-at-home orders, and average temperature

^b^The outcome is counts of domestic violence-related firearm injuries (nonfatal and fatal). Adjusted estimates are from a model that additionally includes: a pre-post dummy for March 2020, COVID-19 cases and deaths, mobility, unemployment, baseline firearm purchasing rates, and stay-at-home orders

*DV* domestic violence, *RR* rate ratio, *CI* confidence interval

### Firearm purchasing and domestic firearm violence

Excess firearm purchasing was positively associated with domestic violence-related firearm injuries in April (RR: 2.60; 95% CI: 1.32, 5.93) and May (RR: 1.79; 95% CI: 1.19, 2.91); these were the months when physical distancing was generally at its peak (Supplementary Fig. 2; Additional file 1). There was no association between firearm purchasing and firearm injuries from domestic violence in June (RR: 1.03; 95% CI: 0.66, 1.52) or July (RR: 0.89; 95% CI: 0.66, 1.12) (Table 2).

### Additional and sensitivity analyses

Results from alternative model specifications were generally similar (Supplementary Tables 2–3; Additional file 1). However, estimates for domestic firearm violence were attenuated and confidence intervals crossed the null when adjusted for state-specific linear trends (Supplementary Table 3; Additional file 1).

## Discussion

There were substantial increases in firearm purchasing and firearm violence in the US during the coronavirus pandemic. We estimated a nationwide excess of 4.3 million firearm purchases from March through July 2020 and 4075 more injuries from interpersonal firearm violence than expected from April through July. These findings are consistent with recent studies and media reports (Kravitz-Wirtz et al., 2021; Sutherland et al., 2021; Bates, 2020; Nass, 2020; Lyons et al., 2021).

Despite concomitant increases in firearm purchasing and firearm violence nationally, the magnitude of the increase in purchasing at the state-level did not explain the magnitude of the increase in non-domestic firearm violence. There was an association between firearm purchasing and domestic firearm violence in April and May; these were the months when physical distancing was at its peak, indicating that risk for domestic firearm violence associated with excess firearm purchasing may have been exacerbated by increased time spent at home and in the context of pandemic-related stressors. However, the estimates for domestic firearm violence were sensitive to the inclusion of state-specific linear trends, suggesting that unmeasured, state-specific, time-varying confounders influenced our findings. Such confounders may include trends in drug and alcohol use or changes in access to domestic violence prevention and intervention services (e.g., access to courts or community-based organizations).

This is the first study, to our knowledge, to estimate the association between firearm purchasing surges and firearm injuries from domestic violence specifically; it is also the first, that we know of, to leverage interstate variation in estimating the association with firearm assault. Previous research has found an association between firearm purchasing spikes and interpersonal firearm injury at the city level (Laqueur et al., 2019) and unintentional firearm deaths at the state level (Levine & McKnight, 2017). Differences in findings may result from different temporal and geographic units of analysis (e.g., cities versus states), exposure measurements (e.g., handgun purchases versus background checks), and outcomes under study (e.g., unintentional versus intentional injuries). Our results may also diverge from those of prior studies due to the context in which the current surge in purchasing occurred.

The summer of 2020 was far from typical, with increases in anxiety, grief, substance use, economic strain, disruptions to daily routines, high-profile instances of police brutality, and a national mobilization against systemic racism, which was accompanied by civil unrest (Torales et al., 2020; Krieger, 2020). Each of these factors may act alone or in combination to increase firearm violence, such that the contribution of firearm purchasing in this context was not statistically detectable. This may help explain why findings from our earlier analysis, with data only through May 2020, found an association between the purchasing surge and violence (domestic violence represents a relatively small proportion of all firearm violence and so is unlikely to have accounted for the results) (Schleimer et al., 2020a). The current study suggests the importance of other contributing factors to the pandemic-related increase in firearm violence and the need for additional research. For example, future research should examine the relationships between violence during the pandemic and job loss and economic support policies; physical distancing and the closure of schools and community organizations; neighborhood social disorganization; civil unrest; and changes to policing.

### Limitations

The current ecological study does not estimate the individual-level risk of firearm violence association with firearm acquisition; prior research suggests that it is high (Studdert et al., 2020). We also do not estimate the risks of firearm access during the pandemic, but rather the risks of *excess* firearm access. Pandemic-related stressors likely increased risk of firearm violence among those with pre-pandemic firearm access, and marginal changes in purchasing, particularly among existing firearm owners, may not have had population impacts on violence. This may partially explain our null findings.

In addition, we have no information on firearm storage practices, whether the excess firearms acquired were those used in violence, or characteristics of purchasers, including how many were new firearm owners. Evidence from California from July 2020 suggests that over 40% of people who acquired a firearm in response to the pandemic were new owners (Kravitz-Wirtz et al., 2021).

There are also data limitations. GVA and NICS data provide imperfect measures of firearm violence and purchasing, respectively. Disagreement between NICS checks and purchased firearms would most likely result from an increase in multiple-firearm transactions during surges in purchasing, which would introduce a conservative bias in estimates of the number of firearms purchased during surges. Further, our estimates are likely conservative as not all sales are accompanied by a background check. In states without laws regulating private party firearm sales, 57% of such sales may occur without a background check; nonadherence is approximately 26% in states with laws regulating private party sales (Miller et al., 2017). Additionally, as GVA data are based on news reports and other public sources, records of firearm violence may reflect undercounts due to incomplete reporting to police and news coverage, perhaps particularly for nonfatal injuries and in recent months, as many local newspapers have lacked resources for reporting or shut down. A recent study, which focused on three cities in 2017, suggests that approximately half of shooting victims known to police were not captured in GVA that year (Kaufman et al., 2020). However, because the GVA database is event based, rather than victim based, it likely provides a more reliable estimate of the number of events and injuries than of specific victim characteristics, including age and gender. Some events might lack sufficient information to make a determination about whether they were DV related or not, resulting in misclassification of DV incidents as non-DV incidents. Such misclassification could make the non-DV results appear more similar to the DV results than they would otherwise be. Despite these limitations, GVA is the most comprehensive real-time database of firearm violence, to our knowledge, and it has been used in prior research (Kim, 2019; Leibbrand et al., 2020). To bias our results, there would need to be similarly-timed differential changes across states in GVA or NICS reporting. Additionally, NICS data are not available at the substate level and are only reported in monthly aggregate, preventing us from examining the relationships at more granular geographic and temporal scales.

Finally, we measure short-term associations and focus narrowly on intentional interpersonal firearm violence. GVA does not provide data on suicide in real time, as it does other types of firearm violence, and examination of unintentional firearm injuries was beyond the scope of the current paper. Despite evidence suggesting that firearm purchasing rates have not returned to pre-pandemic baseline (Federal Bureau of Investigation, 2021), we do not include more recent data because ARIMA forecast errors increase with forecast length. Future research should examine longer-term risks related to the firearm purchasing surge and additional outcomes, including suicide and unintentional injuries, as risk for both types of firearm violence may increase with this surge.

### Broader implications

Although results from the present study generally do not support an association between an acute pandemic-related increase in firearm purchasing and firearm violence at the state level, we estimated a substantial increase in firearm injuries and deaths, suggesting a need for evidence-based and equitable violence prevention efforts. Given the impulsive nature of most firearm violence and the multiple stressors associated with the pandemic, such efforts may include short-term crisis interventions, e.g., extreme risk protection orders (Rivara et al., 2021) and those involving outreach workers or conflict mediators (Corburn & Fukutome, 2021). Strategies to reduce domestic violence in particular may include screening for firearm ownership and intimate partner violence in healthcare settings (including telehealth), improving access to support services and basic resources like quality Internet, and increasing the adoption and use of domestic violence restraining order firearm prohibitions (Evans et al., 2020; Zeoli et al., 2016).

Results of the current study also align with prior research suggesting that firearm purchasing behavior is, at least in part, a social phenomenon driven by macro-level factors. Prior research (Kravitz-Wirtz et al., 2021; Lyons et al., 2021) and media reports suggest that fears regarding personal safety and civil unrest contributed to the current surge in purchasing. For example, of the estimated 110,000 Californians who reported acquiring a firearm in response to the pandemic in July 2020, many cited concern for lawlessness, prisoner releases, the government ‘going too far,’ and government collapse as primary reasons for acquisition (Kravitz-Wirtz et al., 2021). Notably, these concerns may be exacerbated by continuing political violence, such as the insurrection at the US capital on January 6th, 2021 (Leatherby et al., n.d.). While we do not detect a robust association in the current study, a large and growing body of literature ties firearms to increased—rather than decreased—risk of firearm injury (Miller et al., 2002), underscoring the paradox of protection and making surges in firearm purchasing nonetheless potentially concerning for public health. Though the perceived threat itself may be different, researchers have similarly hypothesized that perceived threat responses underlie prior spikes in firearm purchasing related to mass shootings and political events (Studdert et al., 2017). This is consistent with the fact that firearms are, in general, often owned for protection against people (Azrael et al., 2017). Notwithstanding the need for more research on the psychosocial underpinnings of firearm ownership and the specific contexts that motivate firearm acquisition, evidence suggests addressing misperceptions about the health risks and benefits of firearm ownership (Schleimer et al., 2020b) and improving people’s sense of collective trust and security may reduce the burden of firearm violence.

## Conclusion

We found substantial increases in firearm purchasing and firearm violence during the coronavirus pandemic. Our results suggest that state-level excess firearm purchases may have contributed to an increase in firearm injuries from domestic violence during the first months of the pandemic, but that the increase in purchasing did not explain a large increase in non-domestic firearm violence. There is a need for additional research and investment in firearm violence prevention strategies.

## Supplementary Information

**Additional file 1.** Additional Material. Additional Information about Data Sources, Methods, and Results.

## Data Availability

Some of the datasets used and/or analysed during the current study are available from the corresponding author on reasonable request. SafeGraph mobility data are available from SafeGraph but restrictions apply to the availability of these data, which were used under license for the current study, and so are not publicly available. However, SafeGraph data are freely-available to researchers upon request (https://www.safegraph.com/covid-19-data-consortium).

## Abbreviations

DC: District of Columbia
NICS: National Instant Criminal Background Check System
GVA: Gun Violence Archive
ARIMA: Auto-regressive integrated moving average
AIC: Akaike Information Criteria
