# Supplementary material for: Firearm purchasing and firearm violence during the coronavirus pandemic in the United States: a cross-sectional study
Source: Inj Epidemiol. 2021 Jul 5;8:43. doi: 10.1186/s40621-021-00339-5 (PMC8256207; doi:10.1186/s40621-021-00339-5)
Supplement: Supplementary file 1 — Additional file 1. Additional Material. Additional Information about Data Sources, Methods, and Results. [file 40621_2021_339_MOESM1_ESM.pdf]

Additional Material

Firearm purchasing and firearm violence during the coronavirus pandemic in the United States: a cross-sectional study

Julia P. Schleimer, MPH, Christopher D. McCort, MS, Aaron B. Shev, PhD, Veronica A. Pear, MPH, Elizabeth Tomsich, PhD, Alaina De Biasi, PhD, Shani Buggs, PhD, MPH, Hannah S. Laqueur, PhD, MA, MPA, Garen J. Wintemute, MD, MPH

Table of Contents

A) Assessment of Potential Confounders
   a. Supplementary Figure 1. Directed Acyclic Graph (DAG)
B) Additional Information About Data Sources and Variables
   a. Supplementary Table 1. Data Sources and Variable Descriptions
C) Additional Descriptive Data
   a. Supplementary Figure 2. Change in the Percentage of Population Staying Home by State
D) Additional and Sensitivity Analyses
   a. Supplementary Table 2. Supplementary and Sensitivity Analyses, Non-Domestic Firearm Fiolence
   b. Supplementary Table 3. Supplementary and Sensitivity Analyses, Domestic Firearm Violence

A) Assessment of Potential Confounders

Supplementary Figure 1. Directed Acyclic Graph (DAG)

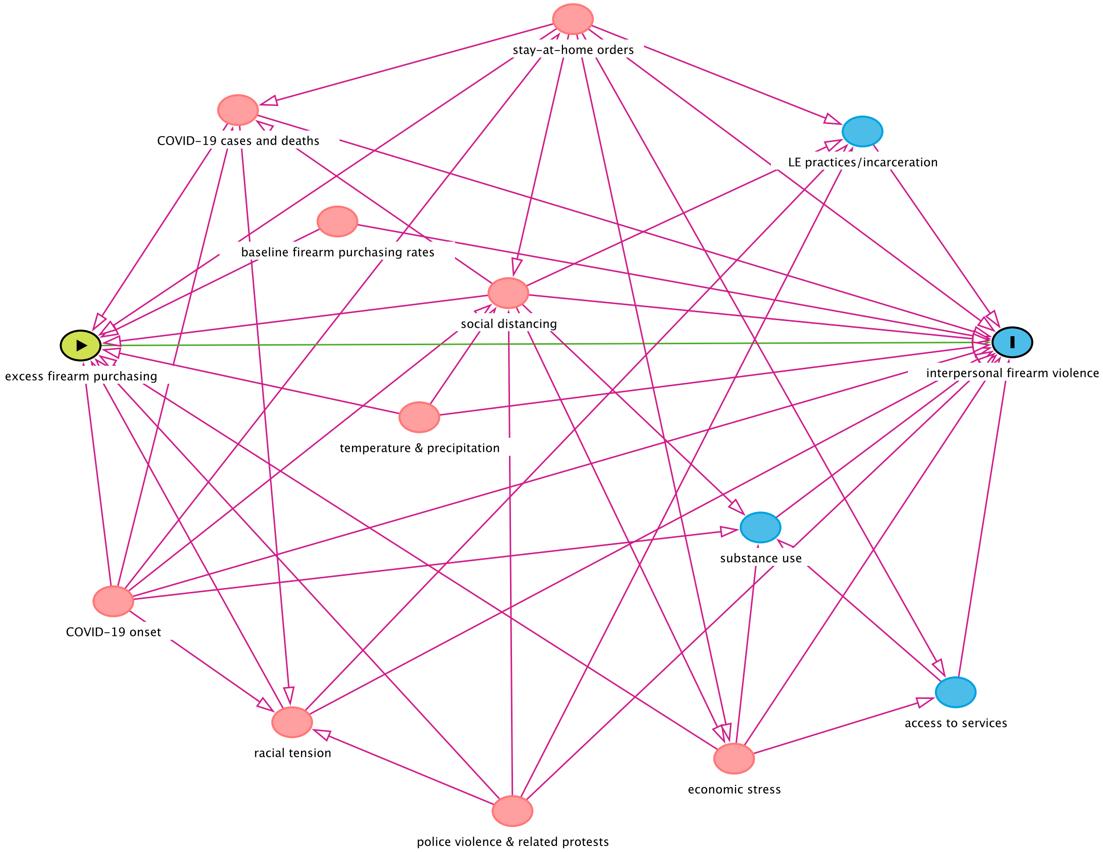

exposure outcome ancestor of outcome ancestor of exposure *and* outcome

causal path biasing path

Note. Relationships are conditional on state fixed effects. We believe coronavirus deaths and cases, social/physical distancing, economic stress/unemployment, and baseline firearm purchasing rates to be stronger confounders than other variables, hence our approach of choosing this limited set of covariates and selecting the rest by comparing Akaike Information Criteria. LE = law enforcement. DAG generated by DAGitty v3.0 available at http://www.dagitty.net/dags.html

B) Additional Information About Data Sources and Variables

Supplementary Table 1. Data Sources and Variable Descriptions

Variable	Source	Description	Notes
Firearm violence	Gun Violence Archive[1]	Nonfatal and fatal injuries from intentional, interpersonal violence events with 1 or more shots fired and 1 or more persons killed or injured	Accessed August 2020
Firearm purchasing proxy	FBI National Instant Criminal Background Check System[2]	Firearm purchase transactions (handguns, long guns, other, and multiple) per population	Accessed August 2020
Unemployment rates	Bureau of Labor Statistics, Local area unemployment statistics[3]	Percentage of the civilian labor force ages 16 and older unemployed	Accessed August 2020
Google searches for racial epithet	Google trends[4]	Trends over time in google searches containing n-word (ending in "er" or "ers");[5] ranked from 0 to 100 based on relative popularity within state over study period[6]	Accessed August 2020 via gtrendsR package (version 1.4.5)
Protesters	Count love[7]	Attendees at protests against racial injustice per population	Accessed August 2020
Incidents of police violence related to George Floyd protests	Crowdsourced database of Police Brutality During the 2020 George Floyd Protests[8]	Incidents of "excessive force, as well as other misconduct, by law enforcement officers during the 2020 protests sparked by the death of George Floyd" per population	Accessed August 2020
COVID cases and deaths	Johns Hopkins University Center for Systems Science and Engineering, time series of cases and deaths[9]	Cumulative number of monthly confirmed COVID-19 cases and deaths per population	Accessed August 2020

Variable	Source	Description	Notes
Temperature and precipitation	PRISM Climate Group, Oregon State University[10]	Average monthly temperature (degrees Fahrenheit) and precipitation (inches)	Accessed August 2020. Bulk download requires latitude and longitude; we used the latitude and longitude of state capitals.
State stay-home orders	New York Times, Mervosh et al., 2020a[11] (version April 20, 2020) and Mervosh et al., 2020b[12] (version August 18, 2020)	Proportion of month state-wide stay-home order was in place	Accessed August 2020
Physical distancing/ mobility	Safegraph[13] aggregated and anonymized smartphone mobility data	Change in percentage of population staying “completely at home” compared to baseline (i.e., average percent of people staying home each day across the seven days ending February 12, 2020)	Accessed September 2020. We adjusted for sampling bias using the recommended post-stratification weighting approach.[14]
All-cause mortality	Centers for Disease Control and Prevention	Monthly counts of deaths per state population, 2019-2020 (updated August 12, 2020)[15] and 2014-2018.[16] We excluded deaths from interpersonal firearm violence and COVID-19 (measured separately)	Accessed August 2020
Population estimates for rate calculations over time	US Census	Annual Estimates of the Resident Population for the United States, Regions, States, and Puerto Rico: April 1, 2010 to July 1, 2019 (NST-EST2019-01)[17]	Data for 2020 extrapolated with cubic regression
Change to state background check laws	RAND State Firearm Law Database[18]	Binary variable indicating change of law classes “background checks” or “permit to purchase” during study period	Accessed September 2020

C) Additional Descriptive Data

Supplementary Figure 2. Change in the Percentage of Population Staying Home by State

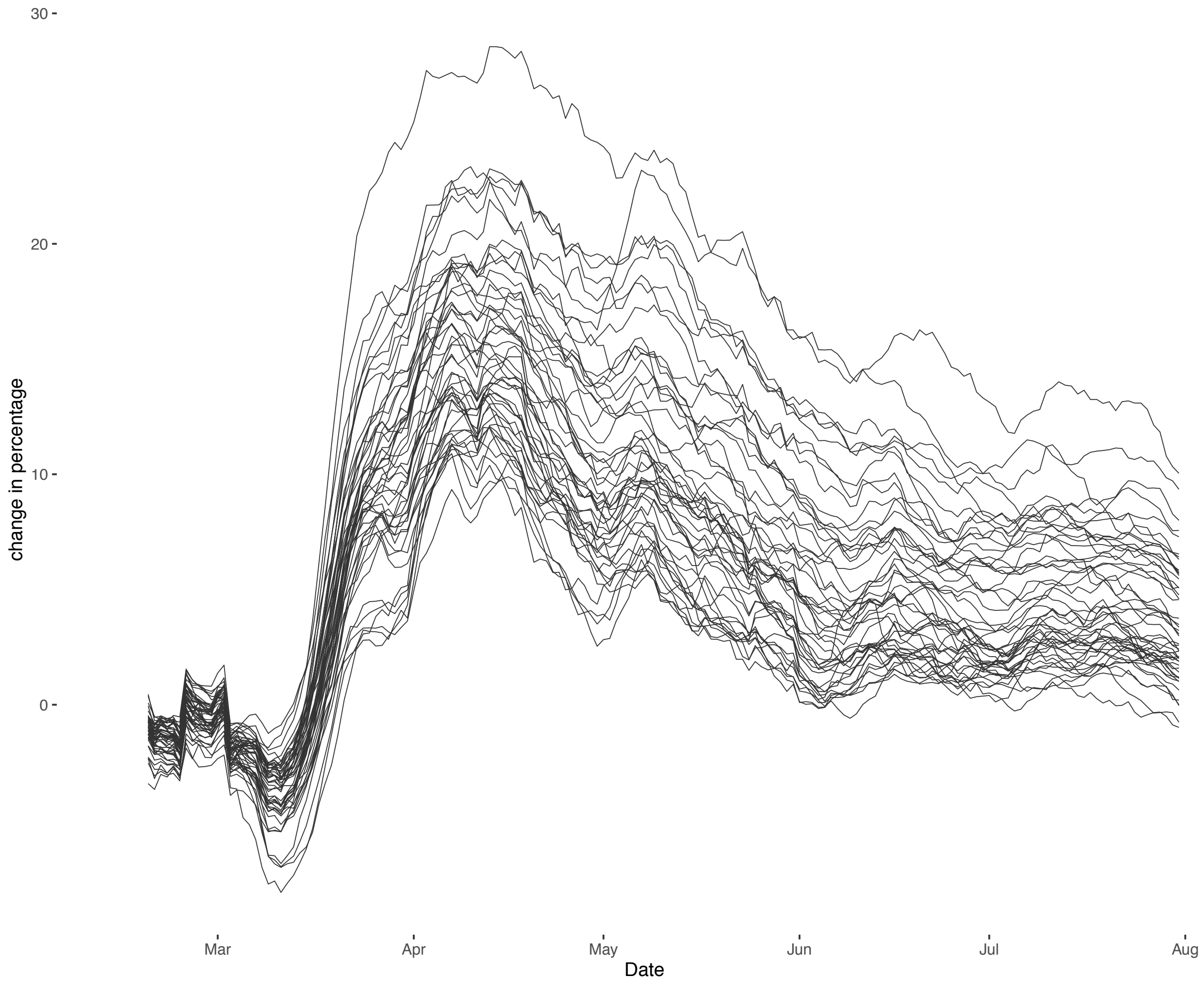

State-wide average change in percentage of population staying home compared to baseline (i.e., the seven days ending February 12, 2020). Trends reflect seven-day moving averages from February 19, 2020 – July 31, 2020. Data Source: SafeGraph

D) Additional and Sensitivity Analyses

Supplementary Table 2. Additional and Sensitivity Analyses, Non-Domestic Firearm Violence

	Events[a]			Injuries per events[b]			State-specific linear trends[c]			Lagged dependent variable[d]			Excluding DC[e]			Controlling for all-cause mortality[f]			Controlling for all hypothesized confounders[g]		
	RR	95% CI		b	95% CI		RR	95% CI		RR	95% CI		RR	95% CI		RR	95% CI		RR	95% CI	
1 excess purchase per 100 population																					
April	0.76	0.51	1.01	-0.13	-0.33	0.04	0.86	0.59	1.13	0.76	0.50	1.02	0.83	0.57	1.06	0.82	0.55	1.10	0.76	0.49	1.03
May	0.97	0.70	1.21	-0.07	-0.19	0.05	1.08	0.81	1.32	0.99	0.72	1.25	1.07	0.82	1.32	0.95	0.68	1.21	1.00	0.72	1.26
June	1.13	0.97	1.34	-0.07	-0.14	0.00	1.16	1.00	1.38	1.11	0.94	1.33	1.09	0.94	1.33	1.08	0.91	1.30	1.11	0.93	1.33
July	1.00	0.86	1.14	-0.04	-0.09	0.02	1.02	0.90	1.17	0.99	0.86	1.13	0.96	0.83	1.09	0.99	0.85	1.13	0.99	0.85	1.13

All models include indicators for state, year, and month; a pre-post dummy for March 2020; COVID-19 cases and deaths; mobility; unemployment; baseline firearm purchasing rates; police violence during the George Floyd protests; stay-at-home orders; and average temperature. Unless otherwise noted, the outcome is counts of non-domestic violence-related firearm injuries (nonfatal and fatal), and results are from negative binomial regression models (with the log of the population as an offset).

[a]The outcome is counts of events involving non-domestic violence-related firearm injuries (nonfatal and fatal).

[b]The outcome is the ratio of non-domestic violence-related firearm injuries (nonfatal and fatal) to events. Results are from a linear regression model.

[c]Results are additionally adjusted for state-specific linear trends.

[d]Results are additionally adjusted for two-month lagged rates of non-domestic violence-related firearm injuries.

[e]The District of Columbia (DC) is excluded.

[f]Results are additionally adjusted for all-cause mortality rates.

[g]Results are additionally adjusted for attendees at protests against racial injustice per population, internet searches for a racial epithet, averge precipitation, and state stay-at-home orders.

RR = rate ratio. CI = confidence interval. b = beta parameter estimate.

Supplementary Table 3. Additional and Sensitivity Analyses, Domestic Firearm Violence

	Events[a]			Injuries per events[b]			State-specific linear trends[c]			Lagged dependent variable[d]			Excluding DC[e]			Controlling for all-cause mortality[f]			Controlling for all hypothesized confounders[g]		
	RR	95% CI		b	95% CI		RR	95% CI		RR	95% CI		RR	95% CI		RR	95% CI		RR	95% CI	
1 excess purchase per 100 population																					
April	2.21	1.13	4.86	0.43	-0.04	1.17	1.76	0.80	4.23	2.55	1.29	5.89	2.66	1.36	6.18	2.74	1.31	6.51	2.80	1.37	6.84
May	1.81	1.29	2.93	0.08	-0.28	0.62	1.32	0.75	2.21	1.77	1.14	2.89	1.82	1.21	3.00	1.71	1.11	2.84	1.86	1.23	3.06
June	1.11	0.76	1.68	-0.05	-0.23	0.13	0.87	0.51	1.28	1.03	0.66	1.51	1.03	0.66	1.51	1.01	0.65	1.47	0.98	0.62	1.43
July	0.97	0.73	1.24	-0.01	-0.17	0.19	0.78	0.56	1.00	0.89	0.66	1.13	0.89	0.66	1.12	0.90	0.68	1.15	0.88	0.65	1.12

All models include indicators for state, year, and month; a pre-post dummy for March 2020; COVID-19 cases and deaths; mobility; unemployment; baseline firearm purchasing rates; and stay-at-home orders. Unless otherwise noted, the outcome is counts of domestic violence-related firearm injuries (nonfatal and fatal), and results are from negative binomial regression models (with the log of the population as an offset).

[a]The outcome is counts of events involving domestic violence-related firearm injuries (nonfatal and fatal).

[b]The outcome is the ratio of domestic violence-related firearm injuries (nonfatal and fatal) to events. Results are from a linear regression model.

[c]Results are additionally adjusted for state-specific linear trends.

[d]Results are additionally adjusted for two-month lagged rates of domestic violence-related firearm injuries.

[e]The District of Columbia (DC) is excluded.

[f]Results are additionally adjusted for all-cause mortality rates.

[g]Results are additionally adjusted for attendees at protests against racial injustice per population, internet searches for a racial epithet, averge precipitation, police violence during the George Floyd protests, and average temperature.

RR = rate ratio. CI = confidence interval. b = beta parameter estimate

REFERENCES

1. Gun Violence Archive. Date accessed: August 2020. Available from: https://www.gunviolencearchive.org/

2. NICS Firearm Checks: Month/Year/State. Federal Bureau of Investigation. Date accessed: August 2020. Available from: https://www.fbi.gov/file-repository/nics_firearm_checks_-_month_year_by_state.pdf/view

3. Local Area Unemployment Statistics Home Page. Date accessed: August 2020. Available from: https://www.bls.gov/lau/

4. Google Trends. Date accessed: August 2020. Available from: https://trends.google.com/trends/?geo=US

5. Chae DH, Clouston S, Hatzenbuehler ML, et al. Association between an Internet-Based Measure of Area Racism and Black Mortality. *PLoS ONE.* 2015;10:e0122963

6. FAQ about Google Trends data - Trends Help. Date accessed: September 2020. Available from: https://support.google.com/trends/answer/4365533?hl=en

7. Count Love. Date accessed: August 2020. Available from: https://countlove.org/

8. Police Brutality During the 2020 George Floyd Protests. Date accessed: August 2020. Available from: https://github.com/2020PB/police-brutality

9. Johns Hopkins University. *COVID-19 Data Repository by the Center for Systems Science and Engineering (CSSE) at Johns Hopkins University*. Date accessed: August 2020. Available from: https://github.com/CSSEGISandData/COVID-19

10. PRISM Climate Group, Oregon State U. Date accessed: August 2020. Available from: https://prism.oregonstate.edu/explorer/

11. Mervosh S, Lu D, Swales V. See Which States and Cities Have Told Residents to Stay at Home. April 20, 2020. The New York Times. Date accessed: August 2020. Available from: https://www.nytimes.com/interactive/2020/us/coronavirus-stay-at-home-order.html

12. Mervosh S, Lee JC, Gamio L, et al. See How All 50 States Are Reopening. June 12, 2020. The New York Times. Date accessed: August 2020. Available from: https://www.nytimes.com/interactive/2020/us/states-reopen-map-coronavirus.html

13. SafeGraph. Shelter in Place Index: The Impact of Coronavirus on Human Movement. Date accessed: September 2020. Available from: https://safegraph.com/dashboard/covid19-shelter-in-place/

14. SafeGraph. Measuring and Correcting Sampling Bias in Safegraph Patterns for More Accurate Demographic Analysis. Date accessed: September 2020. Available from: https://www.safegraph.com/blog/measuring-and-correcting-sampling-bias-for-accurate-demographic-analysis

15. Centers for Disease Control and Prevention. Weekly Counts of Deaths by State and Select Causes, 2019-2020. Date accessed: August 2020. Available from: https://data.cdc.gov/NCHS/Weekly-Counts-of-Deaths-by-State-and-Select-Causes/muzy-jte6

16. Centers for Disease Control and Prevention. Weekly Counts of Deaths by State and Select Causes, 2014-2018. Date accessed: August 2020. Available from: https://data.cdc.gov/NCHS/Weekly-Counts-of-Deaths-by-State-and-Select-Causes/3yf8-kanr

17. US Census Bureau. State Population Totals: 2010-2019. The United States Census Bureau. Date accessed: August 2020. Available from: https://www.census.gov/data/tables/time-series/demo/popest/2010s-state-total.html

18. Cherney S, Morral AR, Schell TL, et al. Development of the RAND State Firearm Law Database and Supporting Materials. Published online September 17, 2020. Date accessed: August 2020. Available from: https://www.rand.org/pubs/tools/TLA243-2.html
